# Crowdsourcing functional cell therapy binders from generative artificial intelligence

**DOI:** 10.1038/s44320-026-00249-y

**Published:** 2026-10-08

**Authors:** Abdul Vehab Dozic, Caleb A Lareau

**Affiliations:** Computational and Systems Biology Program, Memorial Sloan Kettering Cancer Center, New York, NY 10065 USA

**Keywords:** Computational Biology

## Abstract

Generative AI is advancing protein binder design. Kosonocky, Abel and colleagues report on the *Bits to Binders* competition, which tested 12,000 designs in engineered T cells and identified promising candidates for future cell therapies.

**Figure:**
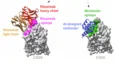

## Teaser

Over thirty years ago, structural biologists established the Critical Assessment of Structure Prediction (CASP) to rigorously assess methods for predicting protein structures from amino acid sequences. Participants submitted predictions before the corresponding experimental structures became publicly available (Moult et al, 1995). This framework established a common standard against which methodological advances could be measured. Its value became particularly evident at CASP14 in 2020, when AlphaFold2 produced predictions approaching experimental accuracy for many targets (Jumper et al, 2021; Pereira et al, 2021). CASP’s independent, prospective assessment enabled the community to recognize the scale of this advance by providing rapid, independent validation. The next generation of CASP-like open competitions is similarly poised to help discern the impact of emerging artificial intelligence (AI)-based methods across several domains of biology, from genomics to protein design to therapeutic discovery. The implementation and results of one such effort, the *Bits to Binders* competition, are described in this issue of *Molecular Systems Biology*, where Kosonocky, Abel and colleagues evaluated 12,000 designed proteins as recognition domains for chimeric antigen receptor (CAR) T cells (Kosonocky et al, 2026).

## Introduction

**Figure 1:**
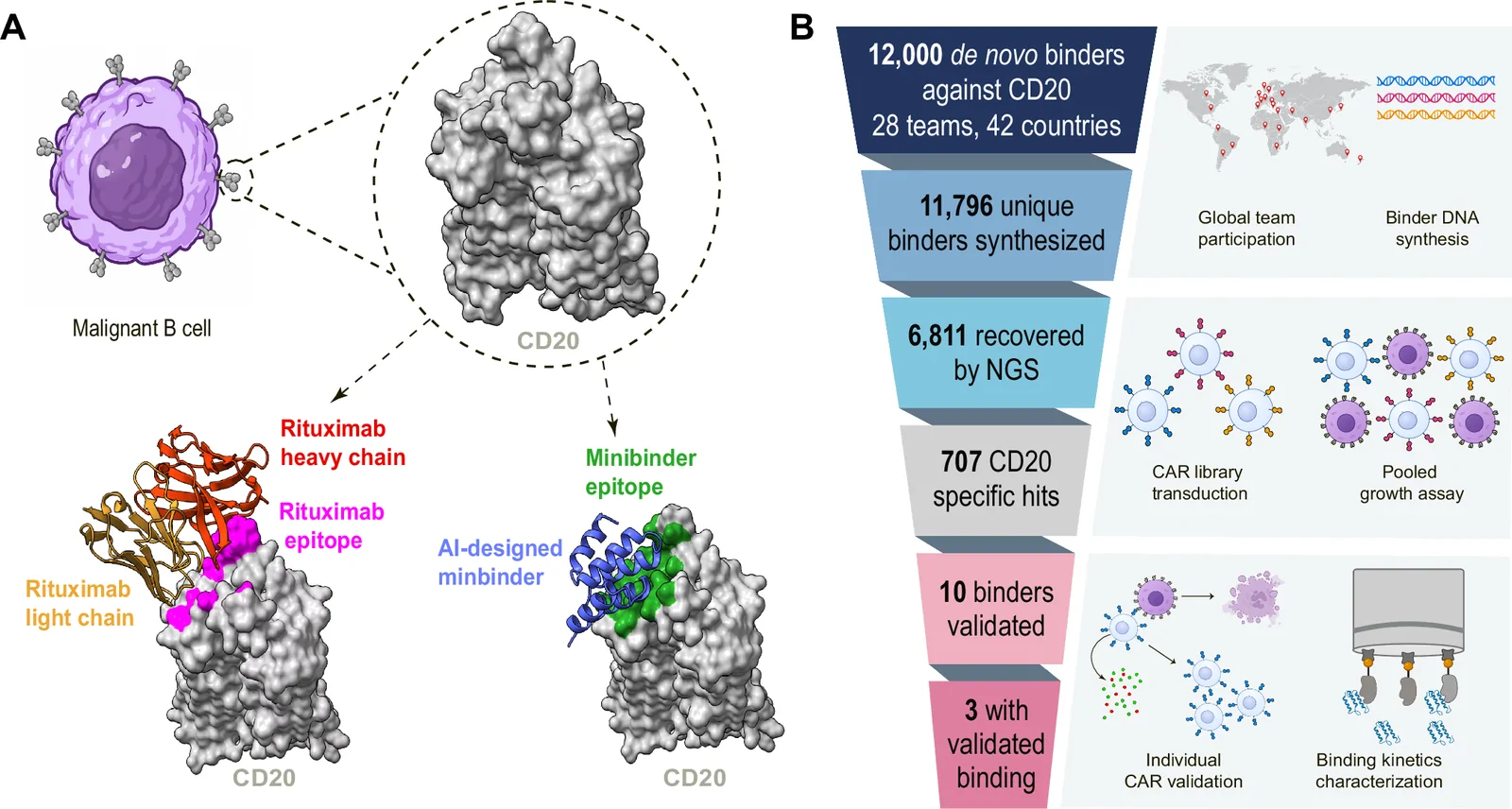
Overview of *Bits to Binders* competition and results. **(A)** Rational of CD20 design, highlighting malignant cell populations expressing the antigen as well as a single-chain variable fragment (scFv) of a therapeutic antibody (Rituximab) and a de novo minibinder identified in this study. **(B)** Schematic of key components of the competition, including the design, testing, and validation of individual binders.

While antibodies have historically been the primary format of therapeutic protein binders, emerging AI methods predominantly produce highly compacted proteins, commonly termed minibinders, that possess many desirable qualities, including well-folded structures, small size (often 65–80 amino acids), and programmable binding interfaces (Koh et al, 2025). Minibinders comprise alpha helices and beta sheets arranged in structures distinct from the antibody-derived single-chain variable fragments (scFvs) conventionally used for antigen recognition in CAR T cells (Fig. 1A). Though the ultimate therapeutic efficacy of minibinders has yet to be determined, their heterogeneous uses have been demonstrated in the academic literature, from biosensors (Quijano-Rubio et al, 2021) to viral neutralization (Cao et al, 2020) to recognition domains in cell therapies (Xia et al, 2024).

Early candidates from de novo protein design have largely been assessed on biophysical parameters, including predicted stability, solubility, and affinity for a chosen target (Chu et al, 2024). Community competitions, including challenges supported by Adaptyv Bio, have begun to compare design workflows through standardized measurements of protein expression and binding affinity. As the field moves toward repurposing binders as therapeutic biologics, these criteria become necessary but not sufficient, since each therapeutic modality carries additional constraints. Extending the framework to therapeutic applications requires benchmarks that establish whether molecular recognition translates into the intended biological response. Bits to Binders establishes an open community effort to assess the functional potential of AI-designed minibinders in CAR T cells. CARs repurpose antigen-binding domains to focus T cells toward specific target antigens expressed on tumor cell surfaces, including CD20, a surface protein expressed by normal B cells and many B-cell malignancies (Fig. 1A).

Twenty-eight teams submitted designs generated using diverse computational workflows. Each design was incorporated into a common CAR scaffold for evaluation in primary human T cells. Productive CAR function requires receptor expression, accessible antigen engagement and appropriate signaling. Recent characterization of de novo CARs identified signaling without a target, inaccessible binding epitopes and off-target activation as distinct constraints for efficacy beyond binding (Chow et al, 2026). These findings underscore the importance of evaluating designed binders in their intended cellular context. Here, the authors used each binder-encoding sequence to track its corresponding CAR T cells in a pooled proliferation screen (Fig. 1B). After growth, 6811 designs met the sequencing recovery threshold; 707 showed significant enrichment in cultures with CD20-positive lymphoma cells relative to cultures without them. Individual testing of ten leading designs confirmed increased proliferation, expansion, and cytokine production for seven compared with unmodified T cells. Four showed significant selective killing in comparisons involving CD20-positive and CD20-negative cell lines. The designs generally underperformed the antibody-derived CAR control, indicating a need for further optimization. Only three showed validated binding in biochemical assays whose conditions differed from the cellular context. Nevertheless, retrospective analyses of the sequences that failed at each step nominate criteria for prospective testing, including screening DNA for high GC content and repetitive sequences, and limiting excessive enrichment of lysine and glutamate in helical structures.

While the overall workflow to aggregate and systematically assess candidate proteins reflects a critical advance, a few caveats signal opportunities to strengthen future competitions. Namely, the pooled screen assessed CAR behavior with or without lymphoma cells, but additional controls, such as isogenic knockouts, are needed to specifically tie activity to CD20. Testing a single receptor scaffold and a single T-cell donor also limits the generalization of sequence features in the binders. Moreover, differences in designed sequences reflect distinct workflows, including sequence selection and refinement, which limit attributing performance to any individual algorithm. Future competitions that expand experimental validation and invite submissions with annotations of designs testing specific pipeline components may better isolate principles for efficacious therapeutic design.

Looking forward, sustaining open competitions will require durable support for experimental testing, independent assessment, and data resources. Long supported by public funding, CASP faced lapses in public funding but secured a one-time private gift in 2025 to sustain the competition. As emerging competitions with more diverse endpoints, including *Bits to Binders*, expand the scope and promise of AI-enabled community benchmarking, open competitions will similarly require sustained investment in the infrastructure necessary to realize biological breakthroughs.
